# The effect of loss of foot sole sensitivity on H-reflex of triceps surae muscles and functional gait

**DOI:** 10.3389/fphys.2022.1036122

**Published:** 2023-01-05

**Authors:** Fangtong Zhang, Mengzi Sun, Feng Qu, Kelsey Lewis, Jung Hun Choi, Qipeng Song, Li Li

**Affiliations:** ^1^ Biomechanics Laboratory, Beijing Sport University, Beijing, China; ^2^ Department of Health Sciences and Kinesiology, Georgia Southern University, Statesboro, GA, United States; ^3^ School of Sports Science and Physical Education, Nanjing Normal University, Nanjing, China; ^4^ Department of Mechanical Engineering, Georgia Southern University, Statesboro, GA, United States; ^5^ College of Sports and Health, Shandong Sport University, Jinan, Shandong, China

**Keywords:** foot sole sensitivity, H-reflex, functional gait, motor control, triceps surae muscles

## Abstract

**Objective:** To investigate the effects of foot sole insensitivity on the outcomes of the triceps surae muscle H-reflex and functional gait.

**Material and Methods:** People with peripheral neuropathy were recruited and divided into two groups: people with more (*n* = 13, 73.3 ± 4.3 years old) or less (*n* = 10, 73.5 ± 5.3) sensitive tactile sensation. Their monofilament testing scores were 9.0 ± 1.5 (range: 7–10) and 2.3 ± 2.4 (range: 0–6) out of 10, respectively. H-reflex of the triceps surae muscles during quiet standing and their relationship with functional gait, 6 min walking distance (6MWD), and timed-up-and-go duration (TUG), were compared between groups.

**Results:** No significant difference was detected for H-reflex parameters between the groups. The less sensitive group showed reduced (*p* < .05) functional gait capacity compared to the other group, 38.4 ± 52.7 vs. 463.5 ± 47.6 m for 6MWD, and 9.0 ± 1.5 vs. 7.2 ± 1.1s for TUG, respectively. A significant correlation (*p* < .05), worse functional gait related to greater H/M ratio, was observed in the less sensitive group, not the other group.

**Conclusion:** Although there was no significant H-reflex difference between the groups, more pronounced tactile sensation degeneration affected functional gaits and their relationship with H-reflex.

## Highlights

1 Foot sole insensitivity changed the proportional relationship of H-reflex.
2 H/M ratio was correlated with function gait among people with foot sole sensation loss.
3 The H reflex of gastrocnemius and soleus is not covariant among people with foot sole sensation loss.

## 1 Introduction

Foot sole sensitivity (transmitted by the smaller type II afferent reflex loop) is more important for posture control than H-reflex (the larger type I afferent reflex loop) (Li et al., 2019). Alterations in cutaneous feedback change gait kinetics and muscular activation patterns (Nigg, 2001; Taylor et al., 2005). People use the foot sole sensitivity to fine-tune their posture and gait (D’Aout, 2019). Age-related decline in cutaneous sensitivity was reported (Perry, 2006). Moreover, foot sole insensitivity associated with peripheral neuropathy (PN) (Prendergast et al., 2004) and other causes has become the second most common deficit in older adults (Mold et al., 2004; Perry, 2006). However, tactile sensory improved after 6 months of Tai Chi practice in people with diabetic peripheral neuropathy (Li and Manor, 2010). Furthermore, stimulation of cutaneous afferent could enhance Hoffman’s reflex excitability (Kamibayashi et al., 2010; Pavailler et al., 2016; Pearcey and Zehr, 2020), improve balance and gait (Lipsitz et al., 2015).

H-reflex and its outcome variables, H-index and H/M ratio, can be used to assess nerve conduction velocity and motor response, respectively (Guiheneuc and Bathien, 1976; Leppanen, 2012). Kneis and others (Kneis et al., 2016) reported that patients with chemotherapy-induced peripheral neuropathy were presented with decreased nerve conduction velocity and motor excitability in the soleus muscle (SOL). The H-index of people with PN exhibited less than that of healthy people and has a negative linear correlation with functional gait, while that of healthy people has not (Zhang et al., 2015b). Although the H reflex cannot distinguish the severity of PN in people with impaired foot sole sensitivity, the lateral gastrocnemius (LG) H-index parameter is reliable (Song et al., 2022) and an excellent method to recognize the people with and without PN (Sun et al., 2022).

H-reflex can be easily elicited in the triceps surae muscles (Lachman et al., 1980) where SOL was the most commonly used muscle for H-reflex studies in humans (Tucker et al., 2005). One of the reasons researchers focused on the SOL was the significant separation between the stimulus thresholds of its M- and H-waves (Capaday, 1997; Tucker et al., 2005). The H-reflex of SOL could help understand the modulation of spinal reflexes in people with PN (Lachman et al., 1980; Schimsheimer et al., 1987). Still, because of the misconception that the H-reflex can only be reliably tested in SOL, the clinical value of the H-reflex has been under-utilized (Burke, 2016). However, PN has a progression route from the distal to the proximal direction, a recent study reported that LG may have better reliability since it is physically more proximal to the SOL (Song et al., 2022). What’s more, the effect of aging on the network deficits of the gastrocnemius muscle (38%) was less than that of SOL (66%) (Ebrahimi et al., 2020). Based on these results, the lateral gastrocnemius may be more sensitive to age-related neuromuscular changes. The H-reflex detected in the lateral gastrocnemius may be more responsive and valuable in older adults with PN.

The H-reflex of soleus and gastrocnemius muscles are similar since they are all innervated by the tibial nerve. The soleus and gastrocnemius muscles are all anti-gravity muscles in function and receive the common synaptic input, but there are different proportions of fast vs. slow muscle fiber and various effects of the inhibitory mechanisms (Duclay et al., 2009; Alrowayeh et al., 2011; Farina and Negro, 2015). The H/M ratio of soleus was greater than that of the gastrocnemius, whether it is standing (Alrowayeh et al., 2011), walking (Makihara et al., 2012), running (Simonsen et al., 2012), or standing with unstable shoes (Friesenbichler et al., 2015). However, despite the anatomical and physiological differences, H-reflex amplitudes of the soleus and gastrocnemius muscles co-vary during standing and walking in healthy adults (Makihara et al., 2012).

Although the H-reflex of triceps surae muscle has been reported, it is not clear whether foot sole insensitivity affects the results of H-reflex on the level of common synaptic input, the synchronized anti-gravity function level, or the individual muscle level. It is indispensable to test the correlation between the soleus and gastrocnemius muscles to see how they synchronize their responses in people with foot sole insensitivity loss.

The purpose of this study was to examine the effects of plantar pressure insensitivity on H-reflex parameters (H/M ratio and H-index) recorded among the triceps surae muscles and their relations with functional mobility. Our primary hypothesis was that people with plantar pressure insensitivity had less H/M ratio and H-index values than their more pressure-sensitive counterparts. The reduction was proportional among the three muscles. The second hypothesis was that the relationships between H-reflex parameters and functional mobility would differ between the two tested groups. Group differences would be the same among the three tested muscles.

## 2 Materials and methods

### 2.1 Participants

The project was approved (Approval No. H20076) by the local Institutional Review Board (IRB) following the Declaration of Helsinki. Twenty-seven participants were recruited from the local community. They signed the informed consent before participation. All participants had physician-diagnosed peripheral neuropathy with etiology commonly unknown.

Potential participants were excluded if they presented with a contraindication to the PAR-Q questionnaire: 1) heart condition; 2) high blood pressure; 3) spinal cord disease; 4) loss of balance because of dizziness or lost consciousness within the past 12 months; 5) bone, joint, or soft tissue problem that could be made worse by becoming more physically active; 6) only do medically supervised physical activity. Four of the twenty-seven participants were excluded from the study because their H-reflex was not observed. The others were divided into two groups according to the plantar pressure detection threshold score (PPDTS): less sensitive to touch (LST, 6 males, 4 females, age: 73.5 ± 5.3 years, height:173.0 ± 1.5 cm, body mass: 96.4 ± 26.3 kg, BMI: 31.7 ± 5.5 kg/m^2^) group: PPDTS ≤6 (Li and Manor, 2010) (2.3 ± 2.4); more sensitive to touch (MST, 6 males, 7 females, age: 73.3 ± 4.3 years, height: 163.8 ± 6.0 cm, body mass: 71.0 ± 15.7 kg, BMI: 26.3 ± 5.2 kg/m^2^) group: PPDTS ≥7 (9.0 ± 1.5).

### 2.2 Procedures

Age, sex, height, body mass, plantar pressure detection threshold, 6-min walk distance (6MWD), timed up-and-go duration (TUG), and H-reflex was recorded. Body Mass Index (BMI) was then calculated with body mass (kg) divided by height (m)-squared.

#### 2.2.1 Plantar pressure detection threshold

The test was performed by the participant lying prone on the treatment table. Sensitivity was assessed with a 5.07-gauge Semmes-Weinstein monofilament (North Coast Medical, Inc., Morgan Hill, CA, United States). Five plantar sites, the heel, midsole, bases of first/fifth metatarsals, and hallux were tested three times (Hondzinski et al., 2010). The monofilament was held perpendicular to the skin and then the participants were asked to report if they felt anything after the filament started buckling. A score of “1” was given when a “yes” response accompanied the detected pressure, whereas a “no” response was given a score of “.” Then the score from each site is added. If the total score is 2 or greater, the site was reassigned to a one. If the site received a total score of 0 or 1, the site was reassigned to a. For example, if the participant says “yes” twice and “no” once at the same site, that site was given an overall score of 1. Vice versa, if the participant said “no” twice and “yes” once, that site would be reassigned a. Then the reassigned scores of both feet were added, and the results would be between 0 and 1.

#### 2.2.2 The six—minute walk test

Two traffic cones were set up 30 m apart along a well-lit hallway. The participant was instructed to walk back and forth around the cones for 6 min without stopping. Then the distance (6MWD) was recorded.

#### 2.2.3 Timed up and go test

A cone was placed 3 m in front of an armed chair. The participant sat back in the chair. When the experimenter said “go,” the participant stood up, walked around the cone, and sat back in the chair. The timer was started on the word “go” and stopped when the participant’s back touched the back of the chair. The three trials were recorded, and the average results were used for further analysis.

#### 2.2.4 H-reflex test

H-reflex tests were conducted with the participants standing upright with their feet shoulder-width apart, upper limbs on the side of the body naturally, ankles at a neutral position, and looking at a cross mark ahead on the wall at eye level. Participants were instructed not to move any part of their body and keep each leg holding half their body weight. The H-reflex was evoked by a 500 microseconds rectangular and monophasic stimulus of the right posterior tibial nerve in the popliteal fossa using a constant current stimulator (Digitimer model DS7A, Digitimer Ltd, Welwyn garden city, England). Surface electromyography (EMG) activity was recorded (Trigno Wireless EMG System; Delsys Inc., Boston, MA, United States) at 2000 Hz, and a common mode rejection ratio of 92 dB at 60 Hz, input impedance >1,015 Ω, a bandwidth filter of 20–450 Hz. Thereinto, the recording electrodes were placed at half the distance between the popliteal fold and the medial malleolus, over the muscle bellies of the SOL, medial gastrocnemius (MG), and LG, respectively. Differential electrodes were placed along the muscle fiber orientations.

A handheld probe (a bipolar stimulation electrode with stainless steel ball electrodes that came with the stimulator) was used to find the optimal stimulation site for the tibial nerve to excite the H-wave at low stimulus intensities. Then a 2-cm in diameter hydrogel cathode (negative electrode) was placed at the selected stimulation site in the popliteal fossa. The anode (5 cm × 8 cm, positive electrode) was placed on the front of the thigh (the long side was parallel to the thigh), a little above the patella after the optimal stimulation site was determined. The cathode and anode electrodes (ValuTrode, Axelgaard, Fallbrook, CA, United States) are reusable hydrogel self-adhesive that came with the stimulator. The skin of the EMG electrode placement site was shaved using disposable razors and wiped clean with alcohol pads before the electrodes were placed. The stimulus intensity started at five milliamps (mA). It increased in small increments (2 mA) every 10 sec until 65 mA, or the maximum M-wave was observed (See Song et al., 2022; Sun et al., 2022 for more details and Song et al. for an exemplar recruiting curve).

### 2.3 Calculation of H/M ratio and H-index

H/M ratio was a normalized H-reflex using the maximum value of H- and M-wave amplitudes (maximum H-wave/maximum M-wave) ×100% (Pierrot-Deseilligny and Mazevet, 2000; Chen and Zhou, 2011).

Normalized nerve conduction velocity was estimated using H-index, which was calculated using the following equation (Scaglioni et al., 2002b; Li et al., 2019; Sun et al., 2022):

$${\left(\frac{Height\;\left(cm\right)}{T_{H\left(ms\right)}-T_{M\left(ms\right)}}\right)}^{2}\times 2$$

where Height was the height of the participant, T_H_ and T_M_ were the timing delays from the stimulus onset to the initiation of M-wave and H-wave responses in ms, respectively.

### 2.4 Statistical analysis

All statistical analyses were performed using SPSS version 24.0 (IBM Inc., Chicago, IL). The H/M ratio and H-index were compared between groups (people more or less sensitive to touch) and muscles by a 2 × 3 Mixed Model Analysis of Covariance (ANCOVA) with BMI as covariance. Results of 6MWD and TUG (distance and duration, respectively) were tested using an ANCOVA with BMI to compare the difference between the groups. An independent sample *t*-test statistically analyzed the anthropometric indexes. For all statistical tests, the significance level was set at *α* = .05. The effect size for each mixed model ANCOVA (partial *η*_*p*_^*2*^) and *post hoc* pairwise comparisons (Cohen’s *d*) were calculated. Small, medium, and large thresholds for partial *η*_*p*_^*2*^ were set at .01, .06, and .14, respectively (Cohen, 1988). Thresholds for Cohen’s *d* were set at .20, .50, and .80 for small, medium, and large effects, respectively (Cohen, 1988). Pearson product correlation coefficient (R) and Adj. R-Square was used to examine linear relationships between the H-reflex of the three muscles with both 6MWD and TUG. The significance of the correlation was determined using the relevant *p-*values. Adj. *R*-values were used to judge the strength of the correlation coefficient. The threshold for very weak, weak, moderate, strong, and very strong correlations were .00, .20, .40, .60, and .80, respectively (Evan, 1996).

## 3 Results

**FIGURE 1:**
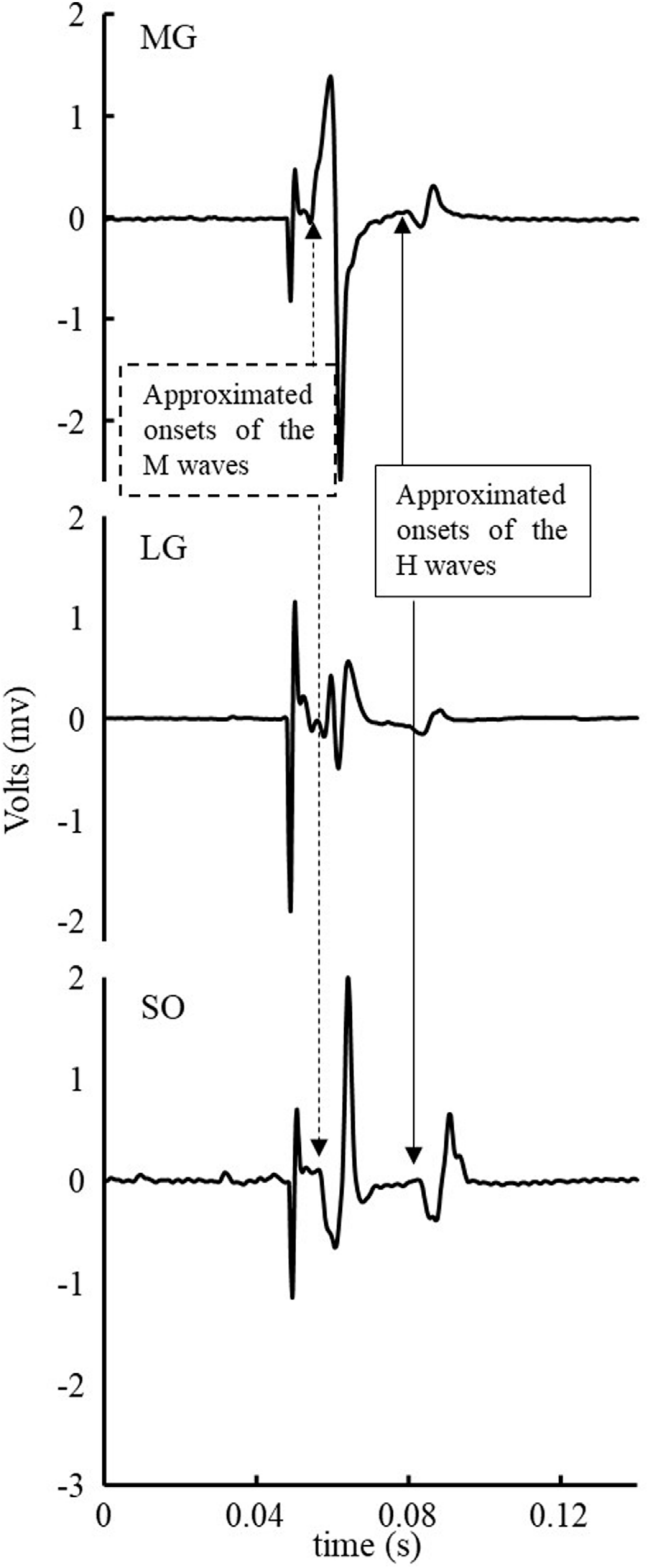
Exemplar EMG signal of a stimulus of triceps surae muscles within 50 milliseconds before the start of stimulus and after the end of the H-wave.

As shown in Figure 1, since there was negligible background EMG of triceps surae within 50 milliseconds before the start of the stimulus and after the end of the H wave when participants were standing, the data of background EMG of the triceps surae was not processed. Background EMG did not interfere with identifying the spatial and temporal H-reflex parameters.

There was no significant difference between the two groups in age. People in the LST group were significantly taller (*p* = .028, *d* = 1.11), heavier (*p* = .017, *d* = 1.21), greater in BMI (*p* = .027, *d* = 1.00), and had lower scores in PPDTS (*p* < .001, *d* = 3.43).

**FIGURE 2:**
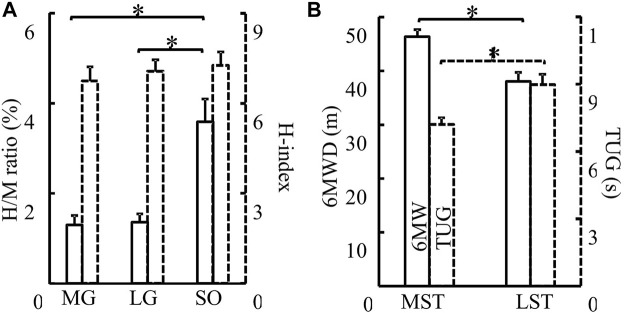
**(A)** H/M ratio (solid) and H-index (dashed) of the MG, LG, and SO muscles were compared. There were no differences between groups in H/M ratio and H-index (*p* > .05). H/M ratio of SO was significantly greater than that of both MG (*p* < .001, d = .232) and LG (*p* < .001, d = .098). No difference in H-index was detected among different muscles **(B)** MST and LST groups were compared for both 6MWD (solid, *p* = .006, partial η2 = .318) and TUG (dash, *p* = .011, partial η2 = .282). The MST group walked farther (6MWD) and fast (TUG) than the LST group. *Indicates a significant difference between muscles (left) or groups (right).

**FIGURE 3:**
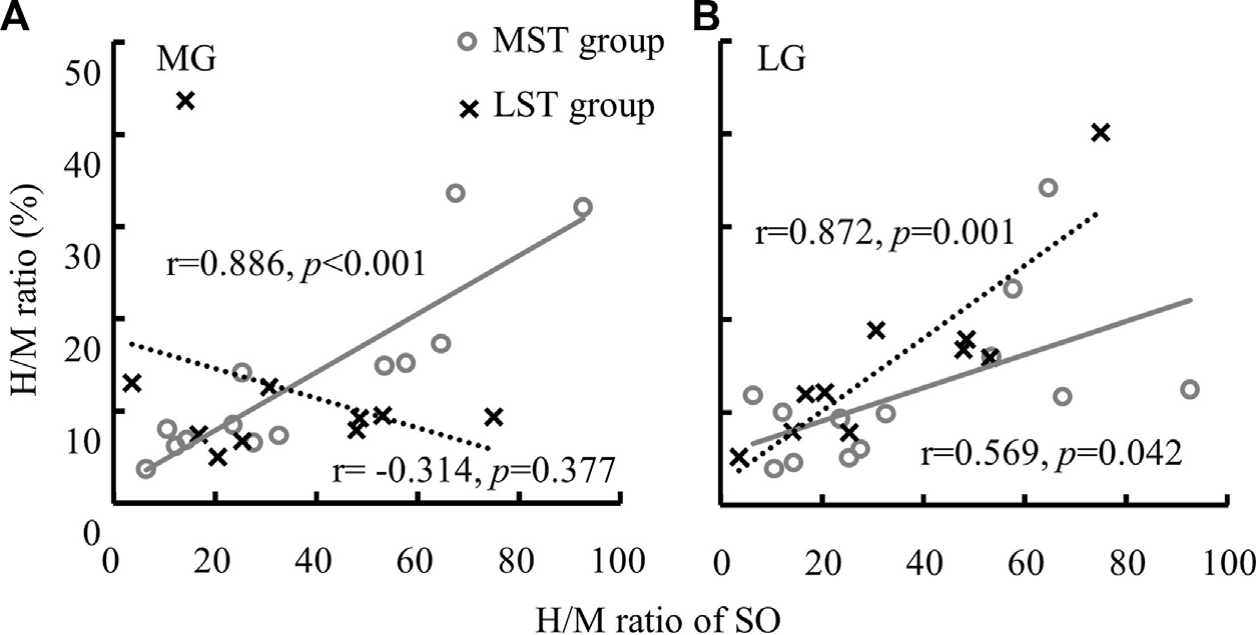
A significant linear relationship between the H/M ratio of SO and MG [**(A)** only in MST group] and the linear relationships between the H/M ratio of SO and LG [**(B)** among both MST and LST groups] were detected.

**FIGURE 4:**
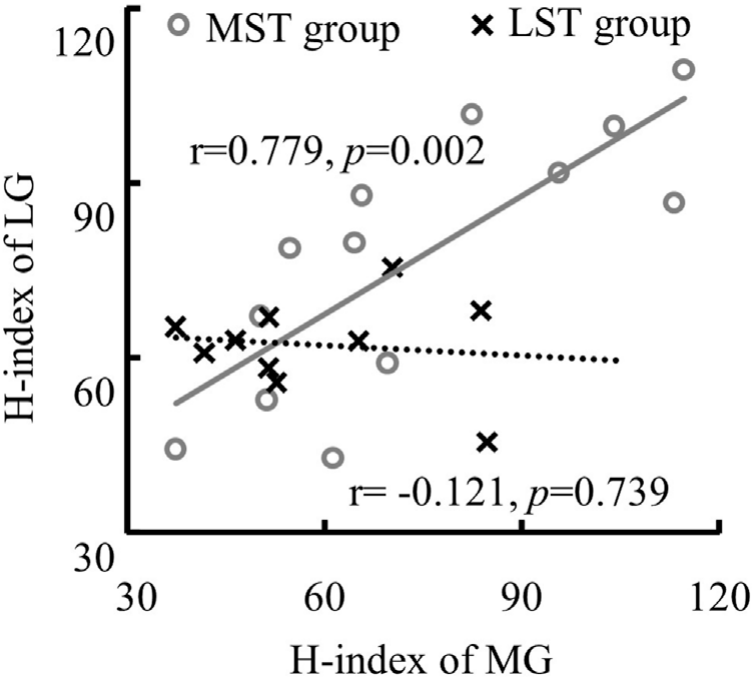
The linear relationship between the H-index of MG and LG with only significant correlation was detected in the MST group.

No significant group by muscle interaction was observed for both the H/M ratio and H-index. No significant difference was observed in the H/M ratio between the MST and LST groups. However, there was a significant difference (Figure 2A for more details) revealed among the three muscles with a large effect size (F_2,40_ = 5.930, *p* = .006, partial *η* ^*2*^ = .229). Specifically, the *post hoc* analysis revealed that the H/M ratio of SOL was significantly greater than that of both MG (*p* < .001, *d* = .232) and LG (*p* < .001, *d* = .098), with small and trivial effect sizes, respectively. No significant difference was observed for H-index between the MST and LST groups, nor among the three muscles. The SOL H/M ratio was only significantly (*r* = .872, *p* = .001, strong) related to that of the LG in the LST group, where the SOL H/M ratio was significantly (*r* = .886, *p* < .001, strong and *r* = .569, *p* = .042, weak, for MG and LG, respectively) associated with that of MG and LG in the MST group. The relationship between MG and LG H/M ratio was not significant in either group. The only significant relationship observed for the H-index was between MG and LG in the MST group (r = .779, *p* = .002, strong) (Table 1; Figures 3, 4 for more details).

**TABLE 1:** Pairwise linear correlation coefficients of H-reflex parameters (H/M ratio and H-index) between triceps surae muscles during standing in both groups.

Group	Muscle	H/M ratio			H-index		
	Pair	*r*	Adj. *R* ^2^	*p*-value	*r*	Adj. *R* ^2^	*p*-value
MST	MG-LG	0.295	0.004	0.328	0.779	0.570	0.002*
	MG-SO	0.886	0.765	<0.001*	0.162	−0.062	0.598
	LG-SO	0.569	0.263	0.042*	0.508	0.190	0.077
LST	MG-LG	−0.246	−0.057	0.493	−0.121	−0.109	0.739
	MG-SO	−0.314	−0.014	0.377	0.580	0.253	0.079
	LG-SO	0.872	0.730	0.001*	−0.286	−0.033	0.424

* indicates the significant linear correlation (*p* < .05).

Significant between-group differences observed for both 6MWD (F_(1,20)_ = 9.309, *p* = .006, partial *η* ^*2*^ = .318) and TUG (F_(1,20)_ = 7.853, *p* = .011, partial *η* ^*2*^ = .282). The LST group walk shorter distances in the 6MWD test and slower in the TUG test (Figure 2B for more details).

**FIGURE 5:**
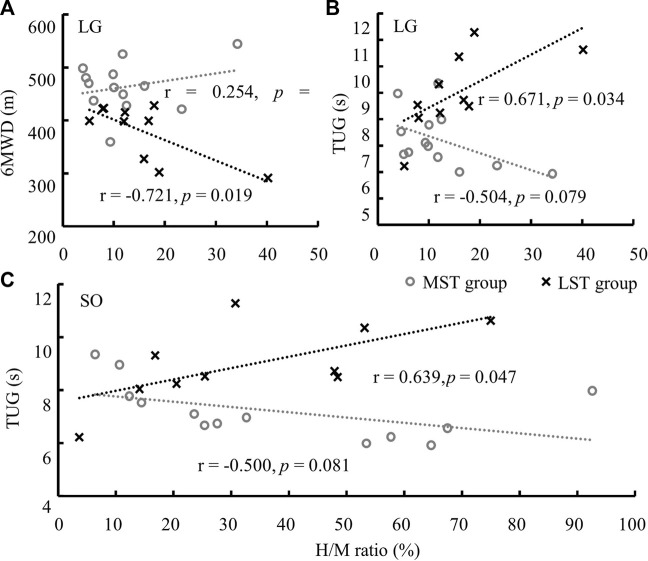
Significant linear relationships were only observed in LST between the H/M ratio of LG and 6MWD **(A)**, and TUG **(B)**, and of SO and TUG **(C)**. Note: the range of SO H/M ratio was much greater than that of the LG.

No significant correlation was detected between the H/M ratio of three muscles with both 6MWD and TUG for the MST group. 6MWD significantly reduced with the increase of LG H/M ratio (*r* = −.721, Adj. *R*-Square = .459, *p* = .019, medium correlation, Figure 5A) for the LST group. However, TUG significantly, but weakly, increased with the increases of the H/M ratios of LG (*r* = .671, Adj. *R*-Square = .382, *p* = .034, Figure 5B) and SOL (*r* = .639, Adj. *R*-Square = .334, *p* = .047, Figure 5C) also for the LST group (Table 2 for more details).

**TABLE 2:** The linear correlation between H-reflex parameters of (H/M ratio and H-index) of the triceps surae during standing and functional gait (6MWD & TUG) in respective groups.

Functional gait	Group	Muscle	H/M ratio			H-index		
			*r*	Adj. *R* ^2^	*p*-value	*r*	Adj. *R* ^2^	*p*-value
6MWD	MST	MG	0.225	−0.035	0.459	0.116	−0.076	0.707
		LG	0.254	−0.021	0.403	0.161	−0.0063	0.599
		SO	0.127	−0.073	0.679	0.224	−0.036	0.463
	LST	MG	0.176	−0.090	0.627	−0.387	0.043	0.270
		LG	−0.721	0.459	0.019*	−0.181	−0.088	0.617
		SO	−0.579	0.252	0.079	−0.024	−0.124	0.947
TUG	MST	MG	−0.317	0.019	0.291	−0.579	0.276	0.038*
		LG	−0.504	0.186	0.079	−0.460	0.140	0.114
		SO	−0.500	0.182	0.081	−0.363	0.053	0.223
	LST	MG	−0.203	−0.079	0.573	0.528	0.188	0.117
		LG	0.671	0.382	0.034*	0.235	−0.063	0.513
		SO	0.639	0.334	0.047*	0.295	−0.027	0.408

* indicates the significant linear correlation (*p* < .05).

**FIGURE 6:**
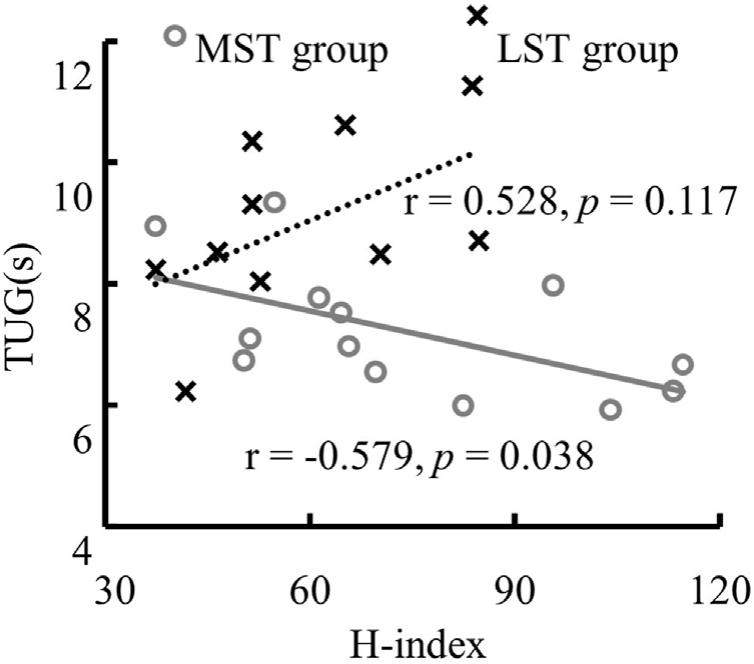
The significant linear relationship between TUG and the H-index of MG in the MST group.

There was only one significant, though weak, negative linear correlation for the MST group (TUG reduced with the H-index of MG, *r* = −.579, Adj. R-Square = .276, *p* = .038) observed among H-index and both 6MWD and TUG for both groups (Table 2; Figure 6 for more details). The H-index of MG decreased over the TUG time in the MST group.

## 4 Discussion

The current study investigated the difference in H-reflex parameters (H/M ratio and H-index) recorded among the triceps surae between MST and LST groups while standing quietly and how these differences related to functional mobility.

The EMG signals of the H-reflex were clean and showed no interference. No significant background EMG was observed that could interfere with identifying the H- and M-wave magnitude and onsets (Figure 1, for example). The low-level voluntary muscle activations of the triceps surae muscles in the tested population agreed with the previous findings (Zhang et al., 2015a; Zhang et al., 2015b). We did not try to eliminate the influence of the background EMG since it is unnecessary. Furthermore, there was no visible crosstalk observed between the three tested muscles (Figure 1 for example), which could be due to the combinations of small detection volume of the electrodes used, relatively large distance between electrodes, and different muscle fiber orientations (electrodes place along with the fiber orientation of each muscle) of the three muscles. In this experiment, the H wave was not observed in four of twenty-seven participants (15%, 4/27). And the proportion of subjects whose H reflex was observed in this experiment (85%, 23/27) was higher than in the previous study (75%, 12/16), in which subjects were elderly and PN (Zhang et al., 2015a). Antidromic action potential and damages to the peripheral nervous system, such as PN-related damage to the axons and myelin of peripheral nerves, among older adults with PN, may be associated with their low H-wave excitation rates (Palmieri et al., 2004; Song et al., 2022). And in young and middle-aged people, the proportion that can stimulate the H-reflex is 100%, while in the elderly and patients with PN, this proportion drops to 79%–90% (Scaglioni et al., 2002a; Chen et al., 2015). This suggested that PN and aging were also reasons for the failure to stimulate the H-reflex (Song et al., 2022).

A smaller H/M ratio was detected with MG and LG than that with SOL, although the hypothesized group difference was not observed. No correlation between MG and LG H/M ratio was observed for both groups. In contrast to the MST group, only the LG H/M ratio was positively correlated with that of SOL in the LST group. In contrast, the LG and MG H/M ratios were positively correlated with the SOL H/M ratio in the MST group. Different from what we have hypothesized, there was no group or muscle difference observed for the standing H-index measures. Furthermore, only one significant correlation was detected for all pairwise relationships in the H-index examined, which was between the MG and LG in the MST group. Contrary to expectations, these results did not support our 1st hypothesis, where we had expected a proportional reduction of the H/M ratio and H-index in the LST group across all three muscles.

Further walking distance (6MWD) and faster walking speed (TUG) were observed with the decreased LG H/M ratio for the LST but not the MST group. Reduced TUG was also related to the decrease in the SOL H/M ratio for the LST group. Increased H-index of MG was related to reduced TUG in the MST, but not LST, group. The average partially supports the last hypothesis that the relationship between H-reflex parameters and functional mobility was different between the two tested groups.

Although there was no difference in the H reflex parameters (H/M ratio and H-index) between the two groups, MST group was better at functional gait than the LST group. The LST group had more severe peripheral nerve degeneration than the MST group. Previous research has reported that foot sole insensitivity causes altered walking gait (Taylor et al., 2005; Alfuth and Rosenbaum, 2012). As the peripheral neuropathy worsens, the patient’s functional movement capacity decreases. However, neural excitability may not decrease linearly, instead, there was an adaptive increase with decreased plantar sensitivity, which may be the result of modulation of the central nervous system due to the long-term peripheral neuropathy.

As shown in Figure 2A, our results were consistent with previous studies showing that the H/M ratio of MG and LG was less than that of SOL (Tucker and Turker, 2004; Makihara et al., 2012). Makihara and others attributed the phenomenon to several factors. The first was different compositions of triceps surae muscle fiber types. SOL has smaller motor units than the gastrocnemius and was also innervated by small-diameter alpha axons. The average innervation ratio of SOL is 180 muscle fibers per motor neuron, however, that of LG was 1,000–200. When voluntary contractions, smaller motor units have lower activation thresholds than the larger motor units, and they would become active before larger motor units based on the “size principle” (Henneman et al., 1965; Mendell and Henneman, 1971). But when electrical stimulation of a peripheral nerve, the motor unit with larger motor axons was more prone to be excited in the reversed activation order (e.g., Hoehler and Buerger, 1981; Stein, et al., 2007). M-waves were generated with direct stimulation to the motor axons. Since the H-wave magnitude follows the “size principle”, the M-wave magnitude instead follows the inversed size principle. SOL with small motor units and small-diameter axons have smaller M-waves and larger H-waves than gastrocnemius muscles, so this may be one reason for the larger H/M ratio of SOL. Second, excitatory postsynaptic potentials (EPSPs), which result in a depolarization of the postsynaptic cell evoked by Iα afferents, were larger in soleus motoneurons than in MG and LG motoneurons in a cat study (Scott and Mendell, 1976). And in humans, the “size principle” explains that small motoneurons with low activation thresholds exhibit large Iα-EPSP amplitudes in SOL (Awiszus and Feistner, 1993). Third, the number of muscle spindles may be greater in the SOL than in the gastrocnemius (Von Voss, 1971). Besides, differential presynaptic and postsynaptic inhibitions may also influence different H-reflex responses between SOL and gastrocnemius (Capaday and Stein, 1989; Stein, 1995; Knikou, 2008). All of these factors could contribute to a higher number of motor units involved in the H wave of the SOL, which may lead to a higher H/M ratio.

The correlation between the H/M ratios of the three muscles in pairs during standing for the MST group agreed with a previously reported study of a healthy population (Makihara et al., 2012). Makihara and others attributed the above observation to the fact that the excitability of the H-reflex pathways was modulated similarly among the three muscles. However, the current study failed to observe the H-reflex magnitude covariation between the gastrocnemius and the SOL in the LST group. The altered synaptic connection could explain the disproportional change of the H/M ratio in the triceps surae muscles due to peripheral nerve injuries (Alvarez et al., 2010). Furthermore, failure to suppress the H-reflex of SOL was highly correlated with postural instability among different pathological populations including PN (Tokuda et al., 1991; Koceja et al., 1995; Hayashi et al., 1997; Kim et al., 2013).

In agreement with the previous results, the present study observed that the LST group walked a shorter distance in 6MWD and took longer in TUG than the MST group (Manor and Li, 2009; Camargo et al., 2015). Furthermore, Camargo and others confirmed that static and dynamic balance deficits were associated with walking speed in people with PN. The slower they walk, the less stable they are likely to be (Camargo et al., 2015). A possible explanation might be that peripheral nerve degeneration leads to increased walking variability and local instability (Manor et al., 2008). The H-index of MG has a significant negative correlation with TUG in the MST group, but that was not consistent with Zhang and co-workers (Zhang et al., 2015b), who observed the H-index of the LG but not MG.

H-reflex might have a predictive value in the diabetic peripheral neuropathy population (DPN) (Millan-Guerrero et al., 2012) and an individual with normal H-reflex rarely has PN (Victor et al., 2001). The present study showed that the functional gait of the MST group was better than that of the LST group, but we failed to detect differences in H-reflex. The differences might mean that both groups had a reduction in the H-index despite the deterioration of plantar sensitivity. A previous study showed that the H-reflex parameter is an excellent method to recognize people with and without PN but not distinguish the severity of the PN with impaired foot sole sensitivity (Sun et al., 2022). Hence, people with PN may exhibit H-reflex abnormalities preceded by a decrease in plantar sensitivity. We assumed that the H-reflex test could be more sensitive to early PN lesions than other types of clinical examinations (Halar et al., 1979).

Consistent with previous studies, the soleus and gastrocnemius muscles have the same modulation in the H-reflex (Kim et al., 2013), and they work in synergy to control posture (Gantchev and Draganova, 1986) and facilitate ongoing movement during walking (Makihara et al., 2012) in healthy people. Given the significant correlation between the H-index of LG and MG, the H/M ratio between MG and SOL in the MST but not LST groups. These results suggested that variations in nerve conduction velocity and excitability level of homonymous monosynaptic reflex due to peripheral neuropathy were not coordinated. That may be explained by the fact that the neuromuscular junctions (NMJs) in both MG and LG were changed due to peripheral nerve degeneration. A rat experiment showed that long-term model DPN lost motor neurons innervating NMJs at very distal sites (Zochodne et al., 2008). In addition, motor neurons innervating the muscle spindle of the soleus and gastrocnemius may deteriorate in varying degrees. There were 0.94 and 0.4 muscle spindles per Gram in the soleus and gastrocnemius muscles, respectively, in the healthy population (Von Voss, 1971), while myelinated afferent fibers alterations due to DPN may originate from muscle spindles secondary terminations (Nardone and Schieppati, 2004). As a result of peripheral nerve degeneration, deterioration of muscle spindles may lead to alteration of the excitability level of the H-reflex loop.

The SOL has a higher proportion of slow-twitch fibers (70%) than the gastrocnemius (50%) (Edgerton et al., 1975). An animal experiment showed that the magnitude of the H-reflex of slow muscle increased compared to fast-twitch fibers. Type 1 alpha-motoneurons were predominantly activated by stimulating afferent Ia fibers (Messina and Cotrufo, 1976). Evidence of a negative relationship between motor unit number estimates and dorsiflexion twitch half-relaxation time may indicate type 2 motor units were lost preferentially in DPN (Allen et al., 2014). Thus, it could be speculated that the gastrocnemius muscle with a higher proportion of fast fibers was preferentially impaired compared to SOL. Thus, the modulation of the H-reflex of gastrocnemius was more sensitive than that of SOL in people with peripheral nerve degeneration. Due to peripheral nerve degeneration, the H-reflex of triceps surae did not co-vary implying foot sole insensitivity altered the modulation of H-reflex among the triceps surae.

The major limitation of this study was that there was no data on the H-reflex of the triceps surae muscles in the lying and walking positions. Even in standing with the ankle in plantarflexion, neural, and dorsiflexion positions, the H-reflex of SOL exhibited differently in a healthy population (Alrowayeh et al., 2011). It is unknown if the relationship between each pair of the three muscles of H-reflex in a lying or walking position would be the same as that during standing in people with PN. Secondly, the sample size was limited. However, our results were robust, and there was enough statistical power for the statistically significant observations. Thirdly, there was no healthy control group in this experiment. The locomotor performance of people with PN may be different from healthy people. In future research, in addition to comparing the difference in PN severity, it is also important to study the difference between people with and without PN. However, these do not affect the conclusion based on the current results.

In conclusion, greater excitability, indicated by a greater H/M ratio, was observed in SOL comparing the gastrocnemius for both groups. No different nerve conduction velocity, measured by H-index, was detected between groups and among muscles. However, foot sole insensitivity changed the proportional relationship of the H/M ratio and the H-index among the triceps surae muscles. It was unexpected to see that less triceps surae excitability was correlated with function gait among the less, but not the more, sensitive to touch group. Thus, the modulation of the H-reflex of gastrocnemius was more sensitive than that of SOL in people with peripheral nerve degeneration. Degenerated foot sole sensitivity reduces functional gaits and their covariation with the H-reflex of triceps surae. This study provides additional information on how peripheral nerve degeneration affects the interaction between the central and peripheral nervous systems. This information helps us to improve the rehabilitation process related to peripheral nerve degradation.

## Data Availability

The original contribution presented in the study are included in the article/Supplementary Material, further inquiries can be directed to the corresponding author.
